# Development of Technology of Restructured Meat Products Using Biotechnological Methods of Transformation of Functional and Technological Properties of Raw Materials

**DOI:** 10.3390/foods14162894

**Published:** 2025-08-20

**Authors:** Alem Beisembayeva, Aigul Tayeva, Irina Chernukha, Berdikul Rskeldiyev, Mamura Absalimova, Zhadyra Imangaliyeva

**Affiliations:** 1 Department of Food Technology, Almaty Technological University, 100 Tole bi Str., Almaty 050000, Kazakhstan; beisembaevaalem@gmail.com (A.B.); berdan_r@mail.ru (B.R.); ustus93@mail.ru (M.A.); i.zhadra.k.90@gmail.com (Z.I.); 2 VM Gorbatov Federal Research Center for Food Systems of RAS, 26 Talalikhina Str., Moscow 109316, Russia; imcher@inbox.ru

**Keywords:** restructured meat products, discarded meat, fortification, bioprotective culture of *Lactobacillus sakei*, microbiological safety of meat products, sustainable food systems

## Abstract

This study developed a technology for restructured meat products (RMPs) from culled cow meat using the bioprotective culture *Lactobacillus sakei* (SafePro B-2, 10^11^ CFU/g) and fortification with L-selenomethionine or zinc citrate. Four variants (Control, SafePro B-2, SafePro B-2 + Se, and SafePro B-2 + Zn) were produced under identical processing conditions and assessed for microbiological, physicochemical, textural, colorimetric, antioxidant, histological, mineral, and amino acid properties. Protein content remained high across all samples (up to 18.7%), while moisture increased by up to 1.4% compared to the control. The Zn-enriched sample showed the greatest cohesiveness and resistance to deformation (*p* < 0.05), with color stability under light exposure improving by up to 12.5%. Despite a reduction in FRAP antioxidant activity (up to 30.8% in buffer extract), the Zn-fortified product exhibited the highest levels of key essential amino acids, including leucine (12.9 mg/g) and lysine (12.6 mg/g). Microbiological analysis confirmed low total aerobic mesophilic counts (≤3.1 log CFU/g), with no detection of *Salmonella* spp. or *Listeria monocytogenes*. Histological evaluation revealed denser and more homogeneous protein matrices in fortified variants. Overall, *L. sakei*-driven bioprotection combined with Se/Zn fortification improved the safety and functional and nutritional characteristics of RMP from low-value beef, supporting sustainable and circular meat production.

## 1. Introduction

Rational utilization of meat resources and development of functional food technology are urgent tasks in the context of global challenges of sustainable development. In the Republic of Kazakhstan, where the total number of cattle is about 8 million, only about 6% are specialized meat breeds, which is significantly lower than the cost-effective indicators adopted in international practice. As a result, much of the meat on the market comes from culled dairy cows, which is characterized by increased toughness, low moisture-binding capacity, unstable microbiological safety, and poor organoleptic performance, requiring integrated biotechnological approaches to processing and resource—efficient valorization strategies. Meat and meat products account for the largest share in the structure of household expenditures—16.9%. The average per capita consumption of meat and meat products was 82.6 kg in 2024 (according to consumption surveys). These figures reflect a stable demand base for meat products and the importance of technological solutions to improve quality and safety in the mass segment [1].

Researchers and food manufacturers around the world are making efforts to improve the quality of meat and meat products by modifying their composition.

According to the review by Gadekar et al. [2], restructured meat products (RMPs) are products made from pieces/fibers fixed into a stable structure to add value to low-grade raw materials. The key techniques are grinding, peeling and forming, and molding; quality is determined by particle size, injection and massaging (extraction of myofibrillar proteins), heating regimen, and coupling system (salt/phosphates, as well as coupling technologies: transglutaminase, fibrin/thrombin, alginate, and vacuum molding). This approach provides predictable texture and yield at an economically feasible cost, which we adopt as the technological starting point for a cull cow product.

Recent studies highlight restructuring as one of the key ways to improve the quality of low-grade raw materials while meeting the demand for functional foods. Restructured meat products are gaining increasing importance as an important component of the meat industry due to consumer interest in their health benefits [3].

Current approaches to meat restructuring include enzymatic and protein-binding technologies that allow precise control over texture, yield, and water-holding capacity. In our study, we focused on controlled fermentation using *Lactobacillus sakei* as an alternative and simultaneously bioprotective strategy to modify the structure and improve product safety [4].

One of the promising solutions is the development of technology for restructured meat products using bioprotective cultures such as *Lactobacillus sakei*. This culture has antagonistic activity against opportunistic microflora, contributes to the microbiological stabilization of the product, and, what is especially important for the processing of tough meat, participates in the natural biological softening of muscle tissue. The softening mechanism is realized through the synthesis of proteolytic enzymes and production of organic acids, causing partial denaturation of proteins and increasing the moisture-binding capacity of raw materials. The high genomic variability and ecological adaptation of *L. sakei* to meat ecosystems allow targeted selection of strains with desired bioprotective and technological traits. As shown in the review by Leroy & De Vuyst [5], lactic acid bacteria are a convenient and safe “tool” for meat systems: they quickly acidify the product, which slows down the growth of unwanted microbes; they moderately break down proteins and fats, which naturally softens the texture and helps form taste and aroma; and many strains additionally produce bacteriocins—natural antimicrobial substances that enhance protection. A number of strains synthesize exopolysaccharides, which improve cohesion and moisture retention. An effective starter culture should work stably under refrigeration and salt conditions, give reproducible results, and meet safety requirements. Based on these principles, in our work we use *Lactobacillus sakei* as a culture that simultaneously increases microbiological safety and gently improves the structure of raw materials from culled cows—without the use of external proteases and high doses of phosphates.

Restructured meat products have many advantages: they are economical, low in fat content, and low in calories [6]. Usually, various additives and binders are used in the development of restructured products [7]. The type and dose of binders, as well as the physicochemical bonding method, critically determine structure, color stability, flavor, and the retention of water and nutrients [8].

Since RM products often consist of a mixture of several meats and sometimes include vegetable components, they can have a wide range of colors, textures, and flavors. To improve the quality of RM products, targeted additives need to be added. The choice of additives and processes is critical in determining the final color, flavor, and texture of the product. For example, the addition of natural colorants such as beet extract or annatto can improve the appearance of the product, giving it a red or pink color that mimics the appearance of traditional meat. However, the production of RM products presents a challenge in terms of achieving proper color while maintaining nutritional value and ensuring consumer acceptance [9].

Texture is crucial for determining the flavor and overall sensory perception of meat products. RM products are carefully processed to reproduce the fibrous structure characteristic of traditional cuts of meat. A study conducted by Ribeiro et al. [10] investigates the effect of different ingredients and processing conditions on the texture of restructured beef. Indicatively, in the study by these authors, the addition of dietary fibers (oat, pea, apple, and inulin; up to 6%) in combination with papain allowed for the control of tenderness, water retention, and yield of restructured meat loaf; the effect depended on the fiber combination and was accompanied by color changes. Based on these observations, in our work we tested an alternative trajectory—controlled fermentation with *Lactobacillus sakei* as a bioprotective and texture-forming tool—and assessed whether it, in combination with Se/Zn fortification, could provide comparable or better-quality performance for raw materials from cull cows without the introduction of external proteases and high doses of binders, while enhancing the microbiological stability and functional value of the product.

The additional enrichment of such products with micronutrients deficient in the diet of the population increases their functional and preventive value. Essential trace elements are necessary for human growth and development, muscle and nerve function, normal cell function, as well as the synthesis of some hormones and connective tissues [11]. Zinc and selenium are important elements in food and are the second most abundant trace elements in the body after iron [12]. They are used as antioxidants and anti-inflammatory agents and are essential for liver function and metabolism. Deficiency of zinc and selenium in the diet or their increased excretion or abnormal metabolism leads to liver diseases [13]. Recent reviews emphasize broad agri-food applications of Zn and Se, including biofortification and oxidative stress protection [14], while in meat systems, the Zn/Se formulation has been shown to enhance antioxidant potential and functional value [15].

In Kazakhstan, there is insufficient selenium and zinc content in soils and water, especially in the East Kazakhstan, Zhambyl, and Aktobe regions [16,17]. This affects the health of the population: selenium deficiency is associated with a high risk of autoimmune thyroiditis, cardiomyopathy, cancer, and reduced immune response [18]. Zinc deficiency leads to stunted growth in children, impaired tissue healing, dermatologic pathologies, and immunodeficiency states [19]. Zinc is essential for normal growth, immune function, and metabolic processes. Its deficiency can cause diarrhea, anorexia, cognitive impairment, skin lesions, hair loss, and delayed puberty [18]. These problems are widespread and are recognized as a priority in the WHO strategy for the prevention of micronutrient deficiencies [20]. Therefore, fortifying mass-consumed foods with physiologically justified doses of Zn and/or Se is viewed as an effective population-level prevention measure [21,22].

The main microbiological hazards that can occur in meat products are the foodborne pathogens *Salmonella* spp., *Campylobacter* spp., *L. monocytogenes*, verocytotoxigenic *Escherichia coli* (VTEC), *Yersinia enterocolitica*, and *Yersinia pseudotuberculosis*, as well as *Staphylococcus aureus*, *Clostridium perfringens*, and *Clostridium botulinum toxins* [23]. Accordingly, bioprotective LAB (including *L. sakei*) are increasingly proposed as an additional hurdle to control *L. monocytogenes* and other hazards, as well as to reduce biogenic amines in fermented meat products [24].

While most existing studies on RMPs rely on exogenous enzymes (e.g., papain or transglutaminase), phosphates, or synthetic additives to modify texture and extend shelf life, the novelty of our approach lies in the use of controlled fermentation with *Lactobacillus sakei* as a dual-purpose agent: to act as a bioprotective culture ensuring microbiological safety and stability and to serve as a natural texture-modifying agent through mild proteolytic activity and organic acid production. This strategy eliminates the need for high concentrations of phosphates or external proteases, which are often viewed with consumer skepticism.

Furthermore, this study pioneers the simultaneous enrichment of RMPs with physiologically significant trace elements—zinc or selenium—addressing region-specific micronutrient deficiencies in Kazakhstan. Unlike previous works where fortification is typically applied to high-value cuts or processed products, we apply fortification to low-value, underutilized meat from culled cows, thereby improving both nutritional quality and economic efficiency.

This study aims to develop two variants of restructured meat products from the meat of cull cows using *Lactobacillus sakei* and enrichment with one of the micronutrients—selenium or zinc. Evaluation of microbiological, physicochemical, structural-mechanical, and sensory characteristics of the obtained samples will determine their potential for industrial production and introduction into the diet of the population, contributing to the solution of problems of sustainable development of food systems.

## 2. Materials and Methods

### 2.1. Materials

Samples of restructured meat products were prepared from muscle tissue of the hip cut. The meat of culled cows of the Holstein breed, 6–7 years old, purchased from the peasant farm “Amantai” (Almaty, Kazakhstan) was used as the main raw material. *Lactobacillus sakei*, strain SafePro^®^ B-2 (Chr. Hansen, Horsholm, Denmark), with a titer of 10^11^ CFU/g, was used as a bioprotective culture. L-selenomethionine (Solgar, Leonia, NJ, USA), with a concentration of 100 µg, and zinc citrate (Solgar, Leonia, NJ, USA), with a concentration of 30 mg of zinc in one capsule, were used as sources of trace elements.

Vacuum tenderization of raw meat was carried out on the ETDU unit equipment, model TUZ. The equipment was developed according to an individual technical specification for the needs of Almaty Technological University and is used for experimental purposes in the development of new meat products.

Phosphate mixture Belfos-90 (manufacturer: Belprodukt CJSC, Gomel, Belarus). The mixture contains sodium pyrophosphates and triphosphates and is designed to improve the functional and technological properties of minced meat.

For salting, a nitrite curing mixture (0.6% NaNO_2_ and 99.4% NaCl) was used, manufactured by Damu-Khimiya LLC (Karaganda, Kazakhstan), 25 kg packaging. The mixture meets the requirements of TR CU 029/2012 and is used to stabilize color, inhibit pathogenic microflora, and extend the shelf life of meat products.

### 2.2. Restructured Meat Product Preparation

The technology of the restructured meat product consists of the following operations: cutting, deboning, fat cutting, chopping, soaking in brine, slicing, shaping, heat treatment, cooling, and storage.

Technological processes were carried out in production facilities with an air temperature not higher than 12 °C and a relative humidity not higher than 75%.

The fatty beef was chopped into pieces weighing 0.2–0.6 kg and syringed with brine in the volume of 20% of the weight of raw material (meat). Water, nitrite salt, and phosphates were used for brine. Further meat raw material was subjected to massaging in a vacuum tenderizer with a rotation speed *n* = 6 rpm for 30 min.

After the tenderization process, the meat was divided into 4 equal parts, one of which was left unchanged as a control sample.

Then activated *Lactobacillus sakei* SafePro B-2 was added to the brine and left to ripen for 16 h at a temperature of 0–4 °C. The study used the SafePro^®^ B-2 starter culture (Chr. Hansen, Denmark) containing the *Lactobacillus sakei* strain CTC494. According to the manufacturer, the viable cell content is at least 1 × 10^11^ CFU/g. The bioculture was activated in warm water (35–37 °C) with the addition of 0.5% glucose for 30 min with stirring. Then, the activated suspension was added to the brine at a rate of 0.5 g of culture per 1 kg of meat, which ensured the addition of approximately 5 × 10^10^ CFU/kg, or 5 × 10^7^ CFU/g of product. Given the use of a standardized culture, additional quantitative verification methods (plate inoculation or spectrophotometry) were not used.

After ripening, the meat was chopped to a size of 10–12 mm, and 10% beef fat was added, chopped in the same way.

The ripened meat was divided into 3 parts: one part was left unchanged (SafePro B-2); L-selenomethionine was added to the second part (SafePro B-2 + Se); and zinc citrate was added to the third part (SafePro B-2 + Zn), as shown in Table 1. Meanwhile, the mineral additives (L-selenomethionine and zinc citrate) were premixed with soy isolate and spices.

The dosages of zinc citrate and L-selenomethionine were determined based on the recommended daily intake (RDI), where zinc is 10–15 mg/day and selenium is 55–70 μg/day (WHO/EFSA). The estimated average consumption is 100 g of the final product per serving. The dosage of each mineral was calculated to provide 20–30% of the RDI per 100 g of product, which guarantees functional enrichment, safety within the upper intake levels, and no negative impact on sensory properties. With a daily intake of selenium = 70 μg/day → 30% per 100 g of product = ~21 μg. With a daily intake of zinc = 10 mg/day → 30% per 100 g = ~3 mg.

These values for the inclusion of selenium and zinc via L-selenomethionine and zinc citrate, respectively, are presented taking into account bioavailability and losses during processing.

Each sample was molded into 300–500 g semi-permeable casings and subjected to heat treatment: drying (60–90 °C, 10–20% relative humidity, for 10 min), roasting with smoke in a thermal chamber (85–105 °C), smoking (70 °C to 50 °C in the center), steam cooking (85–90 °C to 71 ± 1 °C in the center), and cooling to +4 °C with water. The finished products were vacuumed and stored at +2…+4 °C for up to 20 days.

### 2.3. Physicochemical Properties

Determination of the mass fraction of fat was carried out by extraction method using n-hexane, in accordance with ISO 1443:1973 [25], “Meat and meat products—Determination of total fat content.” Determination of the mass fraction of protein was carried out by the Kjeldahl method using a conversion factor of 6.25, in accordance with ISO 937:2023 [26], “Meat and meat products—Determination of nitrogen content—Reference method.” All measurements were performed in triplicate, followed by calculation of the mean value and standard deviation.

### 2.4. Texture Profile Analysis

Determination of loading force by hardness and elasticity indices was carried out on the structure analyzer “Structurometer ST-2.” Sample preparation was carried out before testing. The sample was cut to the size 100 × 20 × 20 mm (length × width × height). The prepared sample was placed in the test field of the texture analyzer and subjected to compression between the lower fixed platform and the indenter “Cylinder Ø36” fixed on the upper movable platform.

The study was carried out according to the method “ST-2 Texture Profile Analysis TPA” on the determination of the loading force on the indenter cylinder Ø36 when it was introduced into the prepared sample to a depth of 5 mm at a speed of movement (introduction) of 0.5 mm/s with the subsequent removal of the cylinder from the sample and its return to the starting point; then, the repeated introduction into the prepared sample to a depth of 10 mm at a speed of movement (introduction) of 0.5 mm/s; then, the indenter returned to the starting point.

The loading force was recorded, and the data obtained were processed using software designed to work with the ST-2 Texture Analyzer (ABC Instruments, Cooper City, FL, USA), using the ST-2 and Algorithm programs (version 4.0; ABC Instruments), following the TPA method. The final measurement result was taken as the arithmetic mean of at least three parallel measurements. The results were processed using Microsoft Excel.

### 2.5. Color

Color characteristics were determined using a Konica Minolta CM-2300d spectrophotometer (Konica Minolta, Inc., Marunouchi, Chiyoda, Tokyo, Japan).

Before conducting the test, the spectrophotometer was prepared for operation and calibrated (zero calibration and white calibration using the plate supplied with the device) in accordance with the operating instructions. Three parallel measurements were taken using the spectrophotometer with color characteristics evaluated in the CIE-L*a*b system, which is a universal color space in Cartesian coordinates. In this system, L* is lightness; a* and b* are color coordinates, where a* is the red–green component of color, and b* is the yellow–blue component of the object’s color, expressed in dimensionless values. The measurement was performed in 3 repetitions. The color stability after 60 min of exposure to incandescent light was also calculated. The following formula was used to calculate the stability:

(1) $$У=\left(1-\left(\frac{\left|L\;1-L2\right|}{3\times L1}+\frac{\left|a1-a2\right|}{3\times a1}+\frac{\left|b1-b2\right|}{3\times b1}\right)\;\right)\times 100\%$$

where *L*1, *L*2 are the lightness values before and after exposure to light;

*a*1, *a*2 are the redness values before and after exposure to light;

*b*1, *b*2 are the yellowness values before and after exposure to light.

### 2.6. Incorporation of Essential Trace Elements

The selenium and zinc content in the samples of the restructured meat product was determined by flame atomic absorption spectrometry using an Agilent Technologies 200 Series AA device, model SpectrAA-280 FS (Agilent Technologies, Inc., Santa Clara, CA, USA).

The tests were conducted in accordance with the requirements of AOAC 999.10-2005 [27], “Lead, cadmium, zinc, copper, and iron in foodstuffs.” Atomic absorption spectrophotometry after microwave digestion, EN 14627:2005 [28], “Foodstuffs—Determination of trace elements—Determination of total arsenic and selenium by hydride generation atomic absorption spectrometry (HGAAS) after pressure digestion.”

Meat samples (0.5–1.0 g) were subjected to wet decomposition in a mixture of concentrated nitric acid (HNO_3_) and hydrogen peroxide (H_2_O_2_) in accordance with the requirements of each standard. Mineralization for selenium analysis was carried out in sealed Teflon autoclave flasks under heating. For zinc determination, an open system was used, followed by evaporation until a clear solution was obtained. After cooling, the mineral residue was brought to the specified volume (usually 25 mL) with distilled water, and the solution was sent for analysis.

Radiation source: hollow cathode lamps (Se: λ = 196.0 nm; Zn: λ = 213.9 nm). The gas mixture was a mixture of acetylene and air.

Nature of analysis: atomization in a flame and measurement of absorption using a standard calibration curve.

The results were expressed in mg/kg of wet product. Each sample was analyzed in triplicate, and the average value was rounded to 0.01 mg/kg. Laboratory quality control included the analysis of a control sample with a known element content.

### 2.7. Antioxidant Activity

The total antioxidant activity of the samples was determined using the Ferric Reducing Antioxidant Power (FRAP) method, in accordance with the methodology proposed in [29], on an SF-2000 spectrophotometer (OKB Spektr, Saint Petersburg, Russia). To prepare the FRAP reagent, 0.3 M acetate buffer (pH 3.6), 10 mM solution of the photometric reagent TPTZ (2,4,6-Tris (2-pyridyl)-s-triazine) (Acros Organics, Shanghai, China), was dissolved in 40 mM hydrochloric acid and 20 mM aqueous solution of iron (III) chloride (PanReac AppliChem, Barcelona, Spain) in a ratio of 10:1:1, respectively. To measure the TAA of the extract, 1.45 mL of freshly prepared FRAP reagent and 50 μL of sample or distilled water for the blank measurement were added to a test tube. The reaction mixture was incubated for 30 min at 37 °C in the dark. After incubation, the samples were centrifuged using a C2204 centrifuge (Liston, Zhukov, Russia) at 3600 rpm for 3 min to precipitate the formed sediment. Then, the optical density was recorded in cuvettes with a distance between the working faces of 1 cm at a wavelength of 594 nm. Measurements for each sample were performed in four replicates. TAA_FRAP_ samples were calculated using a calibration curve (R^2^ > 0.99), which was constructed using quercetin (Sigma-Aldrich, Bangalore, India) in a concentration range of 140–300 μM and expressed in nmol quercetin equivalents/g sample.

### 2.8. Microbiological Analysis

Microbiological assessment of samples of restructured meat products was carried out following current standards and aimed at confirming the sanitary safety and effectiveness of the use of the probiotic culture *Lactobacillus sakei*. Sampling and sample preparation were carried out following ISO 6887-1:1999/2017 [30], “Microbiology of food and animal feeding stuffs—Preparation of test samples, initial suspension, and decimal dilutions for microbiological examination.” The indicators were determined using the following methods:

Colony-forming units of mesophilic aerobic and facultative anaerobic microorganisms (CFU-MA) were determined in accordance with ISO 4833-1:2013 [31], “Microbiology of the food chain—Horizontal method for the enumeration of microorganisms.” The samples were homogenized in a sterile saline solution; serial tenfold dilutions were prepared; and they were seeded on meat peptone agar (MPA). Incubation was carried out at a temperature of 30 ± 1 °C for 72 h. The results were expressed in CFU/g.

The detection of *Salmonella bacteria* was carried out in accordance with ISO 6579:2002 [32], “Microbiology of food and animal feeding stuffs—Horizontal method for the detection of *Salmonella* spp.” Samples weighing 25 g were pre-enriched in selenite broth and then transferred to selective culture media: bismuth sulfite (BS) agar, Endo agar, and xylose-lysine–deoxycholate (XLD) agar. Identification was carried out using standard biochemical tests. The results were expressed as “detected”/“not detected” in 25 g of product.

Analysis for the presence of *Listeria monocytogenes* was performed according to ISO 11290-1:2017 [33], “Horizontal method for the detection of *L. monocytogenes* and *Listeria* spp.” Samples were enriched in selective Fraser broth, then inoculated onto PALCAM agar. After incubation at 37 °C for 24–48 h, suspicious colonies were confirmed by morphological and biochemical characteristics, including catalase activity and β-hemolysis.

The number of mesophilic lactic acid microorganisms was determined according to ISO 15214:1998 [34], “Microbiology of food and animal feeding stuffs. Horizontal method for the enumeration of mesophilic lactic acid bacteria. Colony-count technique at 30 °C.” The culture was carried out on MRS agar (Man-Rogosa-Sharpe, Merck, Darmstadt, Germany) and incubated under anaerobic conditions at a temperature of 30 ± 1 °C for 48–72 h. The colonies were counted, and the results were expressed in CFU/g.

The identification of viable strains of lactic acid microflora, including *Lactobacillus sakei*, was carried out following the requirements of ISO 15214:1998 [34] “Microbiology of food and animal feeding stuffs. Horizontal method for the enumeration of mesophilic lactic acid bacteria. Colony-count technique at 30 °C.” For this purpose, the MALDI-TOF mass spectrometry method was used with the Bruker Microflex LT (Bremen, Germany) device and the Biotyper 3.1 database.

### 2.9. Microstructural Analysis

To assess microstructural changes in restructured meat products with the addition of *Lactobacillus sakei*, selenium, and zinc, a histological study was conducted in accordance with the provisions of regulatory document EN 17644 [35].

Samples were taken from the center of each product (1 × 1 × 0.5 cm cube). Fixation was carried out for 24–48 h in neutral formalin (10% formaldehyde, pH 7.2–7.4), followed by dehydration in increasing concentrations of ethanol (70–96%) and embedding in paraffin.

Paraffin blocks were cut on a microtome (5–7 μm). The sections were applied to slides pretreated with Mayer’s glue, then dried at 37 °C and stained.

Staining was performed with hematoxylin and eosin (H&E) according to Routine Hematoxylin and Eosin Staining Protocols. Staining allowed the morphological organization of muscle, connective, and adipose tissues to be revealed. When necessary, additional staining was performed according to Van Gieson to assess collagen fibers.

Microscopy was performed at ×100 and ×400 magnification using a Leica DM750 light microscope (Leica Microsystems GmbH, Wetzlar, Germany). Images were captured using a Leica ICC50 HD digital camera (Leica Microsystems GmbH, Wetzlar, Germany). Histological evaluation of muscle tissue was conducted by light microscopy after H&E staining according to morphological criteria described in OECD Guidance Document No. 160 and Bancroft’s standard protocols: degree of muscle fiber deformation, amount and distribution of connective tissue, structural homogeneity and presence of pores, signs of protein denaturation and cell destruction, and homogeneity of the restructured matrix.

### 2.10. Moisture and Amino Acid Contents

The mass fraction of moisture was determined by drying the sample with sand at a temperature of 150 ± 2 °C for one hour, according to ISO 1442:2023 [36], “Meat and meat products—Determination of moisture content—Reference method.”

The amino acid profile of the restructured meat product samples was analyzed according to AOAC Official Methods 2018.06. The method is based on acid hydrolysis of protein followed by determination of amino acids by HPLC (high-performance liquid chromatography) after prior derivatization.

A sample suspension (~200 mg) was placed in a vial, 6 M hydrochloric acid (HCl) was added, and it was sealed and kept thermostatic at 110 °C for 24 h in an inert gas (nitrogen) atmosphere to prevent oxidation of sensitive amino acids. After cooling, the hydrolysates were filtered and brought to the specified volume.

Amino acids in the hydrolysate were pre-derivatized with reagents based on ortho-phthalic aldehyde (OPA) or ninhydrin, depending on the system configuration and selected detection. An automated derivatization module at +25 °C was used.

The analysis was performed on an Agilent 1260 Infinity II LC liquid chromatograph (Santa Clara, CA, USA) with fluorescence and UV detectors. Amino acids were separated on a reverse-calibrated C18 column with gradient flow of mobile phase (acetonitrile: water with buffer, pH 6.5). Column temperature was 30 °C, sample volume was 20 μL, and detection wavelength was λ = 338 nm (for OPA derivatives).

Amino acids were identified by retention time of standards. Quantification was performed by the external standard method using a calibration curve.

### 2.11. Statistical Analysis

Statistical analyses were performed using SPSS software (version 24.0; SPSS Inc., Chicago, IL, USA). All experimental values were measured in triplicate, and the results are presented as the mean ± standard deviation. Means within groups were compared using one-way analysis of variance, followed by Duncan’s multiple range test (*p* < 0.05).

## 3. Results and Discussion

### 3.1. Physicochemical Properties

**Figure 1:**
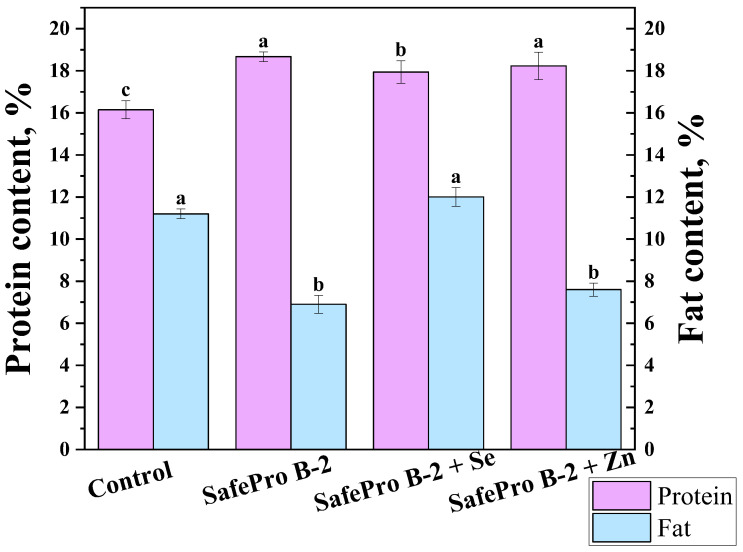
Physicochemical properties of the test samples; control was a restructured product without *L. Sakei* and essential trace elements; letters (a–c) represent significant differences at *p* < 0.05.

The mass fraction of protein in all experimental samples remained at a stable, high level. The highest value was observed in the SafePro B-2 sample (18.67 ± 2.80%), indicating the preservation and even possible activation of the protein structure with the participation of the probiotic culture (Figure 1). Castellano et al. [37] investigated the ability of *L. sakei* to hydrolyze sarcoplasmic and myofibrillar proteins in meat systems. They found that *L. sakei* efficiently cleaves proteins to form peptides and amino acids, which can improve the sensory characteristics of products.

The fat content varied from 6.9 ± 1.0% to 12.0 ± 1.8%. The lowest values were recorded in samples with *Lactobacillus sakei* and zinc, which may indicate an increased ability to retain emulsified fat under heat exposure. In the study by Ameer et al. [38], it was found that inoculating a strain of *L. sakei* during the fermentation of dry sausages leads to a reduction in lipid oxidation, as measured by the level of thiobarbituric acid reactive substances. This indicates improved stability of the fat phase and a potential reduction in fat loss during storage. The reduction in lipid oxidation may be related to the antimicrobial activity of *L. sakei* and its ability to produce compounds that inhibit oxidation processes. The decrease in lipid oxidation may be related to the antimicrobial activity of *L. sakei* and its ability to produce compounds that inhibit oxidation processes. Among such compounds, lactic acid and acetic acid play a key role, which lower the pH of the medium, creating unfavorable conditions for the growth of pro-oxidant microflora that promote lipid peroxidation. In addition, *L. sakei* can synthesize bacteriocins, which inhibit the growth of undesirable microorganisms and thereby reduce microbiologically induced oxidation.

Thus, *Lactobacillus sakei* inoculation contributes to the stabilization of protein and fat fractions of meat products due to proteolytic activity and the ability to produce organic acids and bacteriocins. These metabolites participate in the suppression of pro-oxidant microflora and inhibition of lipid oxidation processes, which provides an increase in the structural and functional stability of the product and prolongs its storage.

### 3.2. Texture Profile Analysis

**Figure 2:**
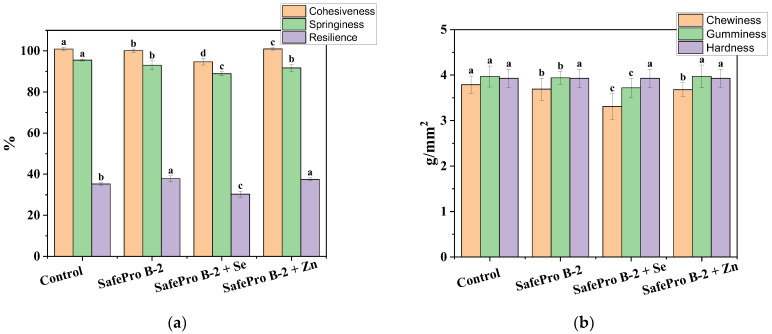
Structural and mechanical properties of the test samples: (**a**) cohesiveness, springiness, and resilience; (**b**) chewiness, gumminess, and hardness; control was a restructured product without *L. Sakei* and essential trace elements; letters (a–d) represent significant differences at *p* < 0.05.

Analysis of the texture profile showed that samples with *Lactobacillus sakei* added demonstrated improved texture characteristics compared to the control group. The highest values of cohesiveness and resistance to deformation were observed in the sample with zinc (SafePro B-2 + Zn), compared to the experimental sample fortified with selenium and the experimental sample SafePro B-2, which may be associated with zinc-induced stabilization of the myofibrillar network (Figure 2a).

Similar results were obtained in a study by Ameer et al. [38], where inoculation with *L. sakei* during the fermentation of dry sausages contributed to an improvement in the textural characteristics of the product, including an increase in cohesiveness. This is due to the enzymatic activity of *L. sakei*, which promotes partial denaturation of proteins and, as a result, increases moisture-binding capacity and forms a denser protein matrix. Additional studies, such as the one by Montero Castillo et al. [39], also confirm that the use of *L. sakei* in combination with functional ingredients such as inulin can improve the textural properties of meat products.

Resilience proved to be a sensitive indicator of texture changes. The values remained high (~37.5–37.8%) in samples with SafePro B-2 and with the addition of zinc, indicating the product’s good ability to recover its shape after deformation. The indicator remained at an acceptable level (~30.3%) even with the addition of selenium (Figure 2a).

All samples showed stable hardness values (~3.93 g/mm^2^), indicating that the protein structure remained intact regardless of composition (Figure 2b). Springiness values ranged from 88.94% to 95.53%. The maximum springiness level was observed in the control group, which corresponds to the typical characteristics of industrially produced ham products. At the same time, the decrease in springiness in the experimental samples, especially in the selenium variant (SafePro B-2 + Se), does not exceed the normative values and can be considered technologically determined (Figure 2a).

The moderate decrease in resilience observed when *Lactobacillus sakei* probiotic culture and biologically active selenium are added may be positively perceived from a consumer perspective. A softer texture improves chewing characteristics, facilitates cutting, and increases the consumer appeal of the product, especially among age groups with reduced chewing ability. In addition, this may indicate a more uniform moisture retention capacity of the protein matrix associated with the action of selenium and the proteolytic activity of *L. sakei*, which contributes to the structural modification of proteins. The addition of *Lactobacillus sakei* and biologically active forms of selenium and zinc does not lead to a deterioration in the structural stability of restructured meat products. All indicators are within the acceptable limits for ham products.

### 3.3. Color

Color is one of the key organoleptic quality indicators of meat products, closely related to the chemical state of myoglobin, the state of the protein–lipid matrix, and exposure to oxygen and light. In the present study, color parameters (L*, a*, b*) of restructured meat products containing *Lactobacillus sakei* with selenium and zinc were evaluated before and after incandescent light source exposure. The measurements were carried out in the CIE L*a*b* system. The results of instrumental studies are summarized in Table 2.

Color stability after exposure for 60 min under incandescent light was also calculated. The data are presented in Table 3.

The results obtained indicate a statistically significant increase in the luminosity index in the samples enriched with trace elements compared to the control, especially in the variant with selenium addition. The control sample showed the greatest increase in lightness after exposure (from 36.87 to 44.39), which, according to the literature, is due to the oxidation of myoglobin and the formation of metmyoglobin, contributing to the lightening of the product. In contrast, the SafePro B-2 + Se and SafePro B-2 + Zn samples showed a decrease in L* after illumination, particularly pronounced in the variant with Se (to 34.36). This indicates a more stable color structure resistant to photo-destructive processes. This color stabilization is supported by foreign studies; thus, Dong et al. [40] found that the introduction of selenium-containing antioxidants into fermented meat products reduced the loss of a* and limited the increase in L* after storage and light exposure.

The most pronounced decrease in a* after exposure occurred in the control sample (-6.31 units), indicating intense degradation of oxymyoglobin. At the same time, the sample with Se showed an increase in redness (18.00 to 19.11), which may be due to the antioxidant protection of the selenium in the myoglobin structure, preventing oxidation to the met form. Similar effects were described in a review by Mancini & Hunt [41] and confirmed in experiments by Luo et al. [42], where fermented meat products with selenium-organic additives showed color retention when exposed to light.

The increase in b* in the control sample (up to 18.50) and the decrease in the samples with *L. sakei* are explained by the degradation of lipid components and the effect of bacteria on Maillard-like reactions. In Se and Zn, this index remained stable after exposure.

In general, all samples show stable and acceptable values of color characteristics, meeting the regulatory requirements for cooked meat products. The data obtained allow us to conclude that the use of the bioprotective culture *Lactobacillus sakei* and selenium- or zinc-containing additives is acceptable without negatively affecting the visual parameters of the product and also provides opportunities for controlled correction of the color profile, taking into account the specified technological conditions, which indicates the prevention of side reactions of oxidation and the possible binding of color-forming substances with microelements.

Calculation of color stability according to L* and a* showed that samples with *L. sakei* + Zn and *L. sakei* + Se had the greatest stability: despite a general decrease in lightness, redness was preserved and even intensified. This confirms that the addition of selenium and zinc contributes to the stabilization of the product’s color parameters under external influences.

### 3.4. Incorporation of Essential Trace Elements

Fortification of meat products with microelements is an effective way to increase their biological value. Selenium and zinc participate in antioxidant defense, immune regulation, and metabolic processes. The use of organic forms—L-selenomethionine and zinc citrate—provides high bioavailability and technological stability during meat processing [43].

The inclusion of selenium and zinc in the formulation is not only due to their biological importance but also due to their pronounced technological effects, especially with regard to stabilization of textural characteristics. According to a pooled review by Wang et al. [44], based on 76 meta-analyses, increased selenium intake is associated with a reduced risk of overall mortality, several cancers (including esophageal, liver, and pancreatic cancers), depression, and Keshan’s disease in children. These data emphasize the relevance of the development of fortified food products that can compensate for nutrient deficiencies in the diet of the population.

Selenium, especially in the form of L-selenomethionine, fulfills protective functions as part of selenoproteins, including glutathione peroxidase and thyroid deiodinases, which are involved in antioxidant defense and regulation of hormonal balance. According to Rayman, Margaret P. [18], selenium deficiency is associated with an increased risk of cardiovascular disease, cognitive impairment, and reduced reproductive potential.

Zinc, according to Roohani et al. [45], is involved in the functioning of more than 300 enzymes, including superoxide dismutase, and is essential for immune defense, tissue repair, and regulation of cell division. Its deficiency manifests as dermatological disorders, impaired immunity, and delayed healing.

In addition to their nutritional importance, both micronutrients have technological advantages in the production of meat products. Selenium inhibits lipid peroxidation, contributing to the preservation of color, flavor, and sensory characteristics. Zinc, as shown by Wu, Q. et al. [46], strengthens the myofibrillar protein matrix and increases the moisture-binding capacity and the cohesiveness of the structure, which is especially important when using restructured and low-grade meat raw materials.

Previously, Dong et al. [40] showed that the addition of selenium and zinc to animal diets or meat product formulations improves color, texture, and oxidation resistance. These findings are confirmed in the present study: enrichment of restructured meat products with a combination of *L. sakei* and micronutrients resulted in improved quality parameters.

**Figure 3:**
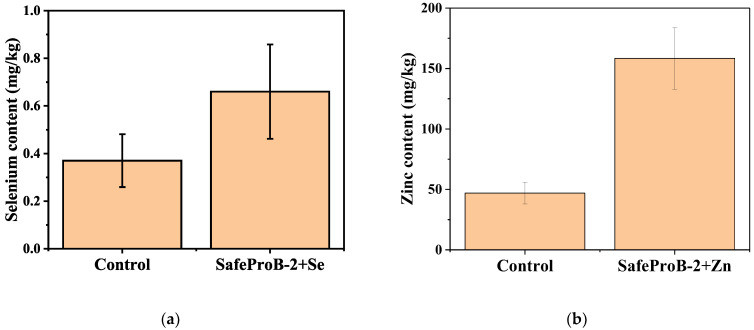
Quantitative content of selenium (**a**) and zinc (**b**); control was a restructured product without *L. Sakei* and essential trace elements; values are shown as mean ± SD.

The results of determining the content of Se and Zn (Figure 3) and their influence on the main consumer and technological characteristics of the product are presented below.

As shown by the fortification study of the test samples, the percentage of selenomethionine increased significantly. Similar results were obtained in a study by Thomson et al. [47], which found that consuming just two Brazil nuts per day resulted in a 64.2% increase in blood selenium levels, comparable to ingesting 100 µg of synthetic selenomethionine. This demonstrates not only a significant increase in selenium metabolites but also an effect on antioxidant enzyme activity. The authors emphasize the high stability and bioavailability (was not assessed) of this form of selenium.

In the experiment by Carlos García-Latorre et al. [48] on the fortification of cereal crops, an increase in Se and Zn concentrations at similar levels was achieved. This confirms the effectiveness of the chosen strategy for meat products.

Thus, selenium and zinc provide both functional and technological improvement of the product and its preventive value as part of a healthy diet.

### 3.5. Antioxidant Activity

Minerals are vital trace elements involved in regulating a multitude of life processes, from enzymatic activity to immunity, bone maintenance, and nerve transmission. Macho-González et al. [15] revealed that minerals such as copper, magnesium, selenium, silicon, and zinc have antioxidant properties and may be good nutritional alternatives for the development of functional meat. The specific values of the total antioxidant capacity of the control and experimental samples are presented in Table 4.

In the case of the control sample and SafeProB-2 + Se, the buffer extract had higher TAA_FRAP_ compared to the ethanolic extracts by 4.12% and 9.77%, respectively. In the case of the SafeProB-2 + Zn sample, the TAA_FRAP_ of the ethanolic extract was higher than the buffer extract by 14.18%. It is important to note that the TAA_FRAP_ of both buffer and ethanol extracts of the sample SafeProB-2 + Se were slightly higher than those of the control product by 2.71% and 8.28%, respectively. While the introduction of zinc into the formulation in SafeProB-2 + Zn led to a significant decrease in the index of antioxidant potential of the product. Thus, the ethanol and buffer extracts of the sample SafeProB-2 + Zn were characterized by a decrease in TAA_FRAP_ by 13.28% and 30.82%, respectively, compared to the control product.

The results obtained confirm the literature data on the positive effect of selenium and zinc on the antioxidant status of meat products. The addition of selenium was accompanied by a moderate increase in antioxidant activity, which is associated with the participation of this trace element in the synthesis of selenoproteins, including glutathione peroxidase, which plays a key role in the neutralization of peroxide compounds [18,44]. Zinc, in turn, is involved in the functioning of many antioxidant enzymes, such as superoxide dismutase, catalase, and glutathione peroxidase, and contributes to the maintenance of cellular redox homeostasis, making it an important element for stabilizing oxidative processes in muscle tissue [45].

Thus, the addition of selenium—especially in its organic form—contributes to the enhancement of antioxidant protection in meat products, which is confirmed by both laboratory measurements and foreign data. The effectiveness of zinc can vary depending on conditions, and its use requires careful selection of form and concentration.

### 3.6. Microbiological Analysis

All samples showed a low level of number of mesophilic aerobic and facultative anaerobic microorganisms (2.4 × 10^2^–1.2 × 10^3^ CFU/g), significantly below the sanitary permissible level for cooked meat products (10^5^ CFU/g according to Regulation (EC) № 178/2002 [49]—General Food Law, ISO 4833-1 [31], and ISO 7218 [50]. This indicates compliance with technological hygiene, effective heat treatment, and the presence of barrier factors.

Notably, the lowest value (1.8 × 10^2^) was recorded in the *L. sakei* + Zn sample, which may indicate a moderate antimicrobial effect of zinc. Similar properties are described in the work of Pasquet et al., where the authors claim that zinc salts, including gluconate, have antimicrobial activity due to the release of Zn^2+^ ions, which disrupt the permeability of bacterial membranes and inhibit metabolic processes [51]. This confirms the decrease in the total microbial count in samples fortified with zinc, as noted in this study.

Microbiological studies of restructured meat products enriched with *Lactobacillus sakei*, selenium and zinc showed compliance with modern sanitary and hygienic requirements. Representatives of *Salmonella* and *Listeria* monocytogenes genus were not detected in any of the samples in 25 g, which corresponds to the requirements of regulations ISO 6579:2002 [32] and ISO 11290-1:2017 [33].

Particular attention should be paid to the quantitative and qualitative presence of mesophilic lactic acid microorganisms (MLMs) including the following strains identified by MALDI-TOF: *Bacillus siamensis*, *Bacillus circulans*, *Bacillus velezensis*, and *Lactobacillus sakei* (in the probiotic variant). Viable strains of *L. sakei* and mesophilic *Bacillus* spp. (*B. siamensis*, *B. circulans*, *B. norneckiae*) were identified in samples using SafePro B-2 and its combinations with microelements, confirming the resistance of the probiotic culture to heat treatment and its possible role in post-process bioprotection. The presence of *Lactobacillus sakei* confirms the viability of the introduced culture after heat treatment and indicates its biosafety.

Thus, it can be concluded that the use of *L. sakei* in the composition of restructured products contributes to microbiological safety without reducing hygienic characteristics. This is consistent with the study of Zagorec & Champomier-Vergès [52], which showed that *L. sakei* is an effective protective culture for meat products due to the synthesis of organic acids and bacteriocins that inhibit the development of undesirable microflora.

### 3.7. Microstructural Analysis

**Figure 4:**
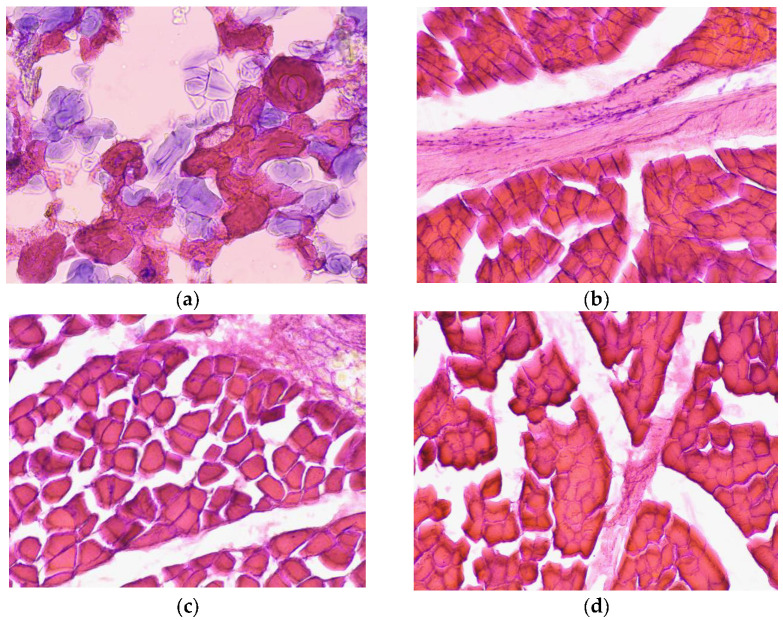
Images of microstructural analysis of control and experimental samples: (**a**) control, (**b**) SafePro-B2, (**c**) SafePro-B2 + Se, (**d**) SafePro-B2 + Zn; control was a restructured product without *L. Sakei* and essential trace elements.

Histological analysis of the control sample obtained from the meat of culled cows revealed characteristic structural features indicating insufficient stabilization of the protein matrix. On sections stained with hematoxylin and eosin, destructured muscle fibers with impaired orientation, pronounced interfibrous gaps, and a fragmented structure are visualized. These signs indicate a low degree of moisture retention, weak protein network organization, and, as a result, a decrease in textural and sensory characteristics (Figure 4a).

Similar morphological features are described in the works of Khvylya et al. [53] and Absalimova et al. [54], where control samples without stabilizing additives were characterized by a loose, heterogeneous structure and multiple fiber defects.

Comparable data are presented in international publications by Luo et al. [42], which showed that control samples without probiotic and functional additives demonstrate microstructural destruction, and the density of the protein framework is significantly lower than in fermented variants.

Huang et al. [55] noted similar degradation of muscle structure in unenriched samples, especially after heat treatment. The authors showed that heat treatment of pork muscle causes sarcomere shortening and muscle fiber condensation, leading to deterioration in texture characteristics, increased cooking losses, and reduced tenderness.

Thus, the morphological state of the control sample confirms the need for texturizing or antioxidant additives that stabilize protein complexes, reduce fragmentation, and form a compact, uniform texture. These conclusions are consistent with the results for structural and mechanical characteristics and moisture content.

Histological examination of the sample with the inclusion of *Lactobacillus sakei* culture revealed marked differences compared to the control sample. Microscopy using H&E staining showed a more orderly arrangement of fibers and a partially remodeled protein matrix structure. The images at magnification show narrowed interfibrous spaces characteristic of muscle structure compaction, smooth fiber contours indicating a reduction in the degree of destruction, and the presence of a homogeneous protein–fat matrix filling the gaps and giving the structure plasticity.

Luo et al. [42] found that the use of *L. sakei* in low-salt fermented sausages contributes to a more uniform microstructure due to the formation of protein–polysaccharide complexes that increase tissue cohesion. Visually, a densification of the structure and a reduction in voids were observed.

Publications by Khvylya et al. [53] emphasize the importance of probiotic cultures in forming a plastic and homogeneous microstructure, especially when using unstable raw materials.

The introduction of *Lactobacillus sakei* leads to the formation of a more organized and dense structure, which is visually confirmed by a decrease in porosity and an increase in muscle tissue cohesion. These changes correlate with the improved textural characteristics obtained earlier in structural-mechanical tests. The morphological picture confirms the promise of using *L. sakei* as a texture-forming and bioprotective component in functional meat products.

Histological analysis of samples enriched with L-selenomethionine, zinc citrate, and *Lactobacillus sakei* bioprotective culture revealed pronounced morphological and functional changes compared to the control group. The images show densely packed muscle fibers forming a homogeneous protein matrix, a minimal number of interfibrous voids indicating effective protein coagulation, clear contours of cell structures without signs of destruction or edema, and the presence of finely dispersed inclusions in the interfibrous spaces (Figure 4b–d).

This morphology indicates stabilization and compaction of the tissue structure, which is due to the synergistic action of *L. sakei* and selenium: the former promotes gentle enzymatic restructuring, while the latter stabilizes desulphated bridges and protects proteins from oxidation.

According to Luo et al. [42], *L. sakei* enhances protein cross-linking and improves their distribution in the product under fermentation conditions. Their micrographs showed a structure similar to the current sample in terms of density and minimal destruction.

The results obtained confirm the synergistic effect of probiotics and selenium on product morphology, which is consistent with foreign data and justifies the technological feasibility of such fortification.

The morphology of the SafePro-B2 + Zn sample indicates the synergistic action of Zn and *L. sakei* in stabilizing the protein–fat matrix and forming a microstructure that is resistant to thermal deformation and degradation.

Sultana et al. [56] showed that the addition of 40 mg/kg of zinc in the form of ZnSO_4_ in the production of chicken meatballs significantly increased the product yield by +6.14% and improved the water-holding capacity (WHC), while the stiffness remained at the control level, which confirms the results of the structural-mechanical study.

Thus, histological analysis of the samples demonstrated the positive effect of *Lactobacillus sakei* and microelement fortification on the morphological characteristics of restructured meat products. The results obtained correlate with improved textural characteristics, demonstrating the technological feasibility and functional effectiveness of the proposed approach.

### 3.8. Moisture and Amino Acid Contents

The analysis showed that the moisture content in the test samples containing *Lactobacillus sakei* was higher than in the control samples: by 1.4% in SafePro B-2 and 0.8% in SafePro B-2 + Zn (Table 5). This may be due to the enzymatic activity of *L. sakei*, which promotes partial denaturation of proteins and, as a result, increases the moisture-binding capacity. This suggests a positive effect of lactic acid bacteria on moisture retention capacity and nitrogen metabolism in the product structure, which is consistent with the findings of Ameer et al. [57], who showed that inoculation with *L. sakei* promotes moisture retention and improves protein quality in fermented meat products. Such findings confirm that the use of *L. sakei* can effectively increase the moisture content in meat products.

The amino acid profile of meat products is a key indicator of their nutritional and functional value. When using non-standard meat raw materials—in this study, meat from culled cows—the formation of a complete amino acid composition requires technological interventions aimed at improving protein quality. One such approach is fortification with trace elements (Se, Zn) in combination with probiotic cultures (*Lactobacillus sakei*), which possess proteolytic activity and can influence the transformation of protein components [58].

Analysis of the amino acid profile demonstrated that enrichment of restructured meat products with *Lactobacillus sakei* culture in combination with L-selenomethionine and zinc citrate improves the protein value of the product. In all experimental samples, an increase in both non-essential and essential amino acids was observed compared to the control (Table 5).

**Figure 5:**
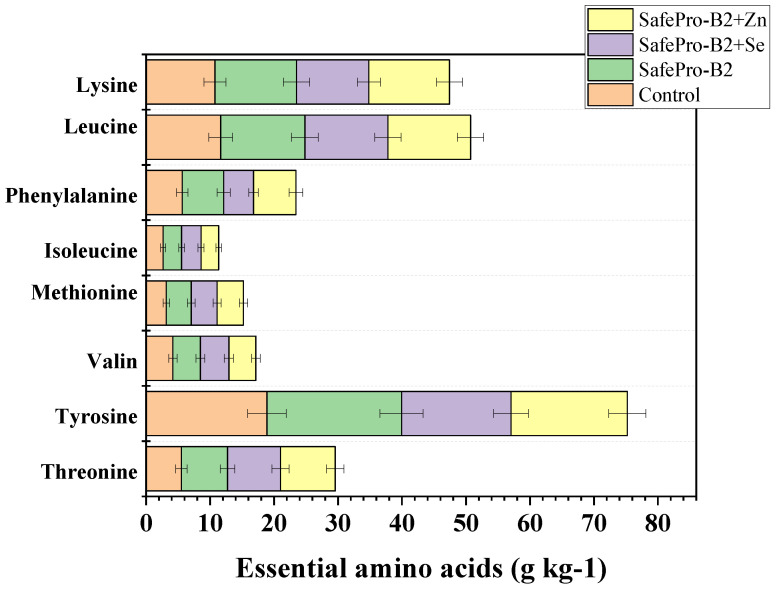
Content of essential amino acids; control was a restructured product without *L. Sakei* and essential trace elements; values are shown as mean ± SD.

Particularly pronounced changes were observed in the samples with zinc addition, which showed the highest levels of leucine, lysine, threonine, and tyrosine (Figure 5).

Samples enriched with *Lactobacillus sakei* and zinc (*L. sakei* + Zn) showed the highest values for most parameters, especially for leucine (12.91 mg/g), lysine (12.60 mg/g), threonine (8.56 mg/g), and tyrosine (18.17 mg/g). This indicates the active participation of zinc in stabilizing protein–mineral complexes and improving the metabolic availability of amino acids, which is supported by the results of other studies.

This amino acid profile indicates increased biological value and potential of the product as a source of quality protein. These data confirm that complex fortification (Zn + probiotic) improves nutrient balance, which is in line with the results of Sultana et al. [56] and similar works on trace elements in meat.

## 4. Conclusions

The results of the study confirmed the effectiveness of using the probiotic culture *Lactobacillus sakei* in combination with organic forms of selenium and zinc in the technology of restructured meat products from culled cows. The use of *L. sakei* ensured the formation of a denser, more cohesive microstructure, which was reflected in increased elasticity and reduced product fragmentation.

Fortification with L-selenomethionine and zinc citrate increased the content of functionally important amino acids (including leucine, threonine, histidine, and arginine), forming a more balanced amino acid profile. It was found that zinc supplementation had a pronounced structure-forming effect, and selenium demonstrated antioxidant activity and a stabilizing effect on the tissue matrix.

Microbiological safety of all experimental samples met the regulatory requirements, while the use of *L. sakei* ensured the preservation of viable lactic acid bacteria and the presence of spore-forming forms of *Bacillus* spp., which confirms the bioprotective potential of the culture. Morphological and histological studies demonstrated a high degree of structural integrity in the enriched samples, especially in the *L. sakei* + Zn variant.

Thus, the proposed technology of fortified meat products provides high safety, improvement of structural and mechanical parameters, and increased nutritional value. The research results show the feasibility of using the biotechnological method of processing and fortification with mineral components of restructured meat products for mass consumption.

## Figures and Tables

**Table 1:** Design and characteristics of each part of the divided raw meat material.

Samples	Supplements	Dosage (Per 1 kg of Meat)	Purpose of the Study
Control	-	-	Sample for comparison
*L. sakei*	*Lactobacillus sakei* (SafePro B-2)	0.5 g	To determine the effect of *L. sakei* on the properties
*L. sakei* + Se	*L. sakei* + L-selenomethionine	0.5 g + 210 μg	Evaluation of the effect of selenium fortification
*L. sakei* + Zn	*L. sakei* + zinc citrate	0.5 g + 100 mg	Evaluation of the effect of zinc fortification

- This indicates the absence of an ingredient.

**Table 2:** Color parameters of meat products samples in the CIE L*a*b* system before exposure.

Sample	L* ± σ	a* ± σ	b* ± σ
Control	36.87 ± 1.46 ^c^	20.92 ± 0.36 ^a^	14.92 ± 1.00 ^c^
*L. sakei*	36.19 ± 0.62 ^c^	20.39 ± 2.01 ^a^	18.24 ± 2.92 ^a^
*L. sakei* + Se	42.48 ± 0.41 ^a^	18.00 ± 0.30 ^c^	16.23 ± 1.36 ^b^
*L. sakei* + Zn	40.61 ± 1.48 ^b^	19.28 ± 2.13 ^b^	17.92 ± 0.72 ^b^

Control was a restructured product without *L. Sakei* and essential trace elements; values are shown as mean ± SD; letters (a–c) represent significant differences at *p* < 0.05.

**Table 3:** Color parameters of meat product samples in the CIE L*a*b* system after exposure.

Sample	L* ± σ	a* ± σ	b* ± σ	Color Stability, %
Control	44.39 ± 2.30 ^a^	14.61 ± 0.43 ^c^	18.50 ± 0.49 ^a^	76.07
*L. sakei*	41.25 ± 1.11 ^b^	15.55 ± 1.21 ^c^	16.26 ± 1.79 ^b^	82.50
*L. sakei* + Se	34.36 ± 0.74 ^c^	19.11 ± 0.72 ^a^	17.71 ± 0.71 ^b^	84.74
*L. sakei* + Zn	35.63 ± 0.93 ^c^	17.43 ± 0.47 ^b^	16.43 ± 0.50 ^b^	88.60

Control was a restructured product without *L. Sakei* and essential trace elements; values are shown as mean ± SD; letters (a–c) represent significant differences at *p* < 0.05.

**Table 4:** Total antioxidant capacity.

Treatment	TAA_FRAP_, nmol-eq, Quercetin/g Sample					m ± SD
Control	Ethanol	214.72	211.42	215.8	212.32	213.57 ± 2.04
	Buffer	233.42	234.55	234.78	234.99	234.43 ± 0.70
SafeProB-2 + Se	Ethanol	231.36	231.94	229.45	232.29	231.26 ± 1.27
	Buffer	237.62	241.36	243.16	240.96	240.78 ± 2.31
SafeProB-2 + Zn	Ethanol	185.68	183.71	182.55	188.81	185.19 ± 2.74
	Buffer	160.52	161.80	162.26	164.17	162.19 ± 1.51

Control was a restructured product without *L. Sakei* and essential trace elements.

**Table 5:** Moisture and amino acid contents.

	Control	SafeProB-2	SafeProB-2 + Se	SafeProB-2 + Zn
Moisture content, %	66.5 ± 6.7 ^b^	67.9 ± 6.8 ^a^	64.3 ± 6.4 ^c^	67.3 ± 6.7 ^a^
Amino acids (g kg^−1^ protein)				
Substituted amino acids				
Asparaginic acid	20.15 ± 3.22	25.62 ± 4.09	23.79 ± 3.80	23.74 ± 3.79
Glutamic acid	27.58 ± 4.41	30.14 ± 4.82	30.81 ± 4.93	27.36 ± 4.37
Asparagine	-	-	-	-
Histidine	5.37 ± 0.86	5.17 ± 0.83	5.75 ± 0.92	5.52 ± 0.88
Serine	7.79 ± 1.24	8.63 ± 1.38	8.93 ± 0.18	8.60 ± 1.37
Cystine	0.89 ± 0.14	0.85 ± 0.14	1.17 ± 0.18	0.95 ± 0.15
Glutamine	-	-	-	-
Arginine	8.65 ± 1.38	10.17 ± 1.63	10.75 ± 1.72	10.81 ± 1.73
Glycine	12.33 ± 1.97	14.42 ± 2.31	13.31 ± 2.12	15.44 ± 2.47
Alanine	16.22 ± 2.59	19.92 ± 3.18	18.94 ± 3.03	19.84 ± 3.17
Essential amino acids				
Threonine	5.51 ± 0.88	7.21 ± 1.15	8.29 ± 1.32	8.56 ± 1.37
Tyrosine	18.87 ± 3.02	21.06 ± 3.37	17.12 ± 2.73	18.17 ± 2.91
Valine	4.18 ± 0.66	4.31 ± 0.69	4.45 ± 0.71	4.22 ± 0.67
Methionine	3.16 ± 0.51	3.89 ± 0.62	4.04 ± 0.64	4.12 ± 0.65
Isoleucine	2.66 ± 0.42	2.89 ± 0.46	3.03 ± 0.48	2.75 ± 0.44
Phenylalanine	5.64 ± 0.90	6.48 ± 1.04	4.66 ± 0.74	6.65 ± 1.06
Leucine	11.66 ± 1.86	13.15 ± 2.10	12.99 ± 2.07	12.91 ± 2.07
Lysine	10.77 ± 1.72	12.74 ± 2.04	11.31 ± 1.81	12.60 ± 2.01
TOTAL:	161.49 ± 25.84	186.69 ± 29.87	179.39 ± 28.70	182.29 ± 29.16

Control was a restructured product without *L. Sakei* and essential trace elements; values are shown as mean ± SD; letters (a–c) represent significant differences at *p* < 0.05.

## Data Availability

The original contributions presented in the study are included in the article. Further inquiries can be directed to the corresponding author.

## Abbreviations

The following abbreviations are used in this manuscript:

RMPs	Restructured Meat Products
WHO	World Health Organization
VTEC	Verocytotoxin-producing Escherichia Coli
H&E	Hematoxylin and Eosin
OECD	Organization for Economic Co-operation and Development
HPLC	High-performance Liquid Chromatography
TBARS	Thiobarbituric Acid Reactive Substances
TAA_FRAP_	Total Antioxidant Activity by Ferric Reducing Antioxidant Power Method
SOD	Superoxide Dismutase
WHC	Water-holding Capacity
